# TLR2/4-mediated NF-κB pathway combined with the histone modification regulates β-defensins and interleukins expression by sodium phenyl butyrate in porcine intestinal epithelial cells

**DOI:** 10.29219/fnr.v62.1493

**Published:** 2018-12-06

**Authors:** Xiujing Dou, Junlan Han, Qiuyuan Ma, Baojing Cheng, Anshan Shan, Nan Gao, Yu Yang

**Affiliations:** Institute of Animal Nutrition, Northeast Agricultural University, Harbin, China

**Keywords:** sodium phenylbutyrate, b-defensins, inflammatory cytokines, signaling pathway

## Abstract

**Background:**

Host defense peptides (HDPs) possess direct antibacterial, antineoplastic, and immunomodulatory abilities, playing a vital role in innate immunity. Dietary-regulated HDP holds immense potential as a novel pathway for preventing infection.

**Objective:**

In this study, we examined the regulation mechanism of HDPs (pEP2C, pBD-1, and pBD-3) and cytokines (IL-8 and IL-18) expression by sodium phenylbutyrate (PBA).

**Design:**

The effects of PBA on HDP induction and the mechanism involved were studied in porcine intestinal epithelial cell lines (IPEC J2).

**Results:**

In this study, the results showed that HDPs (pEP2C, pBD-1, and pBD-3) and cytokines (IL-8 and IL-18) expression was increased significantly upon stimulation with PBA in IPEC J2 cells. Furthermore, toll-like receptor 2 (TLR2) and TLR4 were required for the PBA-mediated upregulation of the HDPs. This process occurred and further activated the NF-κB pathway via the phosphorylation of p65 and an IκB α synthesis delay. Meanwhile, histone deacetylase (HDAC) inhibition and an increased phosphorylation of histone H3 on serine S10 also occurred in PBA-induced HDP expression independently with TLR2 and TLR4. Furthermore, p38-MAPK suppressed PBA-induced pEP2C, pBD-1 pBD-3, IL-8, and IL-18 expression, but ERK1/2 failed to abolish the regulation of pBD-3, IL-8, and IL-18. Moreover, epidermal growth factor receptor (EGFR) is involved in PBA-mediated HDP regulation.

**Conclusions:**

We concluded that PBA induced HDP and cytokine increases but did not cause an excessive pro-inflammatory response, which proceeded through the TLR2 and TLR4-NF-κB pathway and histone modification in IPEC J2 cells.

There are now global voices calling for solutions to the antibiotic resistance problem to guarantee the quality and safety of livestock products and human health. It is projected that 10 million people could die of infectious diseases caused by bacteria, viruses, or fungi by 2050 if effective measures are not taken (1). Such a devastating event should never occur. Therefore, efforts are now urgently needed to formulate a proper strategy for developing new range of antibiotics.

In multicellular animals, plants and insects, Host defense peptides (HDPs) are not only naturally produced but also involved in immunomodulatory and adjuvant functions in the immune cells to boost immune response of the organisms (2); HDPs are also expressed by the host as an antibiotic to protect against potential invading pathogenic microbes via their unique physical properties and membrane-permeabilizing antibacterial mechanisms of action, making drug resistance difficult (3). Recently, the role of HDPs in innate and adaptive immunity is being increasingly appreciated. As an important first line of defense, HDPs are mostly expressed in the epithelial cells of the digestive, respiratory, or urogenital tracts. More than 30 HDPs, including the β-defensin and cathelicidin genes, have been reported to date in pigs (4, 5); indeed, these HDPs include, but are not limited to, β-defensin 1 (pBD1), pBD2, pBD3, pBD129, and epididymis protein 2 splicing variant C (pEP2C), which are present in a wide range of porcine tissues (6).

The hypothesis that HDPs synthesis are induced by some small molecules or dietary compounds which not alter an excessive inflammatory response, this fact will be a promise of preventing and controlling inflammatory response and related infective diseases (7). Butyrate is a short-chain fatty acid (SCFA) naturally produced by colonic bacteria fermentation, and sodium butyrate is capable of inducing HDP expression without affecting the expression of IL-6 enhancing disease resistance in piglets via HDAC inhibition (8). This mechanism is supported by an increased phosphorylation of histone H3 on serine S10 and the activation of the IκB kinase complex, which also leads to the activation of NF-κB. Moreover, both NF-κB and histone acetyltransferase p300 support the enhanced induction of hBD2 expression (9). However, due to the special cheese-like, rancid odor of sodium butyrate, the use is limited among some animals.

Sodium 4-phenylbutyrate (PBA), an aromatic SCFA, is a HDAC inhibitor known for inducing favorable effects on many pathologies, including cancer and pulmonary tuberculosis (10, 11). Indeed, PBA plays an immunomodulatory or anti-inflammatory role. Some studies have focused on cathelicidin antimicrobial peptide (CAMP)-inducing gene expression by PBA in various tissues, and the underlying molecular mechanism of CAMP gene expression has been resolved; interest in this research area is steadily increasing. Previous studies have focused on cathelicidin antimicrobial peptide (CAMP) gene expression induced by PBA in various tissues, however, current research trend focused on induce CAMP expression depends on the vitamin D receptor (VDR) pathway (12) and mitogen activated protein kinase (MAPK) signaling, coupled with PBA-regulated HDP expression displays the gene specific regulation and tissue specificity (13). To date, there is no data regarding how PBA controls HDP expression and exerts its immune defense ability on porcine cells. Herein, we initially show that HDP genes are expressed in porcine intestinal epithelial cells and are enhanced by PBA, but we also show that there is no effect on IL-6 levels. Our results demonstrate that PBA induces HDP expression via the toll-like receptor (TLR) pathway. This process is supported by the phosphorylation of NF-κB p65 independent of myeloid differentiation primary response gene (MyD88) and an IκB α synthesis delay process; this phosphorylation leads to NF-κB activation. PBA possesses a strong ability to inhibit HDAC and enhance the phosphorylation modification of histone H3 on serine S10 in IPEC J2 cells. Thus, we provide novel insights into the regulation of HDP gene expression and evaluate the role of PBA in the innate and adaptive immunity of IPEC J2 cells.

## Materials and methods

### Reagents and antibodies

Sodium phenylbutyrate (purity above 98%) was purchased from Sigma (St. Louis, MO, USA). SB203580 (p38 MAPK inhibitor) and PD98059 (MAPKK inhibitor) were both purchased from Beyotime (Shanghai, China). Gefitinib was purchased from MedChem Express (Trenton, New Jersey, USA). We used rabbit mAb phospho-NF-κB p65 (Ser536, Cell Signaling Technology, Beverly, MA, USA), anti-IκB (SC-371, Santa Cruz Biotechnology, Dallas, TX, USA), anti-β-actin (13E5, Cell Signaling Technology), and secondary horseradish peroxidase (HRP)-conjugated anti-rabbit IgG (4970, 7077, Cell Signaling Technology, Beverly, MA, USA). Dimethylsulfoxide (DMSO) was purchased from Sigma (St. Louis, MO, USA).

### Cell culture

The IPEC J2 cell lines, porcine intestinal epithelial cell lines originally derived from the jejunal crypt of a neonatal piglet, were cultured in DMEM/F12 medium (Gibco, Carlsbad, CA, USA) supplemented with 8% (vol/vol) fetal bovine serum (FBS, Bioind), 5 μg/L ITS (Sciencell, Carlsbad, CA, USA, Cat: 0803), 5 μg/L epidermal growth factor (Sciencell, Carlsbad, CA, USA, Cat: 10504), and 1% penicillin/streptomycin (100 U/mL and 100 mg/mL) (V900929, Sigma, St. Louis, MO, USA) at 37°C with 5% CO_2_.

### Cytotoxicity measurement

The MTT dye reduction assay was used to determine the cytotoxicity of PBA on the IPEC J2 cell lines. Briefly, the IPEC J2 cells were seeded into a 96-well cell culture plate (Corning, NY, USA) with complete DMEM/F12 medium and were grown overnight at 37°C in 5% CO_2_ in a humidified incubator. The cells were washed twice with phosphate buffer solution (PBS), and then fresh DMEM/F12 medium containing different concentrations of PBA was added and incubated for 24 h. Next, 10 μL of MTT (0.5 mg/mL) was added and then the cells were incubated for another 4 h at 37°C. Subsequently, 100 μL of DMSO was added to dissolve the formazan crystals that formed. Finally, the Optical Density (OD) was measured using a microplate reader (TECAN GENios F129004, Austria) at 490 nm.

### RNA isolation and quantitative real-time PCR

Total RNA was isolated with the TRIzol reagent (Ambion Life Technologies, Carlsbad, CA, USA), and almost immediately the cDNA was synthesized using a reverse transcription kit (RR037A, Takara, Ostu, Japan) according to the manufacturer’s instructions. PCR was performed using a SYBR^®^ Premix Ex Taq™ (Tli RnaseH Plus) (RR420A, Takara, Ostu, Japan) on a 7,500 real-time PCR system (Applied Biosystems, Carlsbad, CA, USA). Each sample reaction was run in duplicate on the same plate. The gene-specific primers are presented in Table 1 (14–16). The mRNA expression levels were determined using the 2^−ΔΔCt^ method with β-actin as a reference.

**Table 1:** List of primers used for qRT-PCR

Target gene	Sequence (5’-3’)	Reference/accession
pBD-3	Forward: GAAGTCTACAGAAGCCAAAT	a
	Reverse: GGTAACAAATAGCACCATAA	
pEP2C	Forward: GTTGACCTGGGAGCCAAAG	c
	Reverse: GCACAGATGACAAAGCCTCA	
pBD-1	Forward: CCGCCTCCTCCTTGTATT	MF925344.1
	Reverse: GGTGCCGATCTGTTTCAT	
IL-6	Forward: TGGCTACTGCCTTCCCTACC	b
	Reverse: CAGAGATTTTGCCGAGGATG	
IL-8	Forward: CTGGCTGTTGCCTTCTTG	b
	Reverse: TCGTGGAATGCGTATTTATG	
IL-18	Forward: ACTTTACTTTGTAGCTGAAAACGATG	b
	Reverse: T TT AGG TTC AAG CTT GCC AAA	
TLR2	Forward: TCACTTGTCTAACTTATCATCCTCTTG	b
	Reverse: TCAGCGAAGGTGTCATTATTGC	
TLR3	Forward: AGTAAATGAATCACCCTGCCTAGCA	b
	Reverse: GCCGTTGACAAAACACATAAGGACT	
TLR4	Forward: GCCATCGCTGCTAACATCATC	b
	Reverse: CTCATACTCAAAGATACACCATCGG	
NF-κB1(p50)	Forward: CTCGCACAAGGAGACATGAA	b
	Reverse: ACTCAGCCGGAAGGCATTAT	
NF-κB3(p65)	Forward: TGTGTAAAGAAGCGGGACCT	c
	Reverse: CACTGTCACCTGGAAGCAGA	
β–actin	Forward: GGCTCAGAGCAAGAGAGGTATCC	c
	Reverse: GGTCTCAAACATGATCTGAGTCATCT	

a (14); b (15); c (17); The sequences of pBD-1 in Table 1 are available through GenBank (http://www.ncbi.nlm.nih.gov/nuccore/) under the accession numbers listed above.

### HDAC activity detection

The HDAC activity assay was performed using the amplite™ fluorometric HDAC activity assay kit (AAT Bioquest^®^, Sunnyvale, CA, USA) according to manufacturer’s protocol. Briefly, the IPEC J2 cells were cultured in a 12-well tissue culture plate overnight at a density of 1×10^5^ cells/wells. The cells were treated in duplicate with increasing concentrations of PBA (0–8 mM). A well-characterized HDAC inhibitor, trichostatin A (TSA), was used as a positive control. The cell pellets were harvested after 24 h and homogenized in ice-cold RIPA lysis buffer (P0013B, Beyotime, Shanghai, China) containing the complete protease inhibitor, phenylmethylsulfonyl fluoride (PMSF) (ST506, Beyotime, Shanghai, China). The protein concentration of cell lysates was measured using the trace nucleic acid protein analyzer (Implen, Germany), and the cell lysates were diluted into an appropriate range, which containing equivalent amount of the protein in the assay buffer. Then, 50 μL of the HDAC Green™ substrate working solution was added to each well, and the plate was incubated at room temperature for 45 min. The fluorescence intensity at Ex/Em = 490/525 nm was monitored. The fluorescence was detected in the blank wells with buffer only, which was used as the background and was subtracted from the values determined for the wells subjected to the HDAC Green™ reactions. All the fluorescence readings are expressed in the relative fluorescence units (RFU), and each experiment was performed in triplicate.

### Western blot analysis

For the immunoblot analyses, the total protein was extracted from the cytoplasm of the IPEC J2 cells, and the samples were denatured in 4×SDS-PAGE loading buffer (40 mM Tris-HCl, PH 8.0, 200 mM DTT, 4% (v/v) SDS, 40% (v/v) Glycerol and 0.032% (v/v) Bromophenol Blue) (No. 7173 Takara, Ostu, Japan) and boiled for 10 min. The denatured proteins were separated using 12% SDS-PAGE and were transferred onto a PVDF membrane (0.45 μM) (Millipore, Boston, Massachusetts, USA). The membrane was blocked in Tris Buffered Saline with Tween (TBST) (10 mM Tris, 100 mM NaCl, 0.1% Tween 20) with 5% (w/v) nonfat milk powder for 1.5 h. After washing with TBST for three times, the blocked membranes were incubated with the primary antibodies overnight at 4°C in TBST and were then washed three times followed by incubation with the corresponding HRP-linked secondary antibodies (1:2500) for 1 h at room temperature. The membranes were washed three times, and bound antibodies were detected using an ECL plus detection system (P1010, Applygen, Beijing, China). The expression of each protein was normalized to that of β-actin.

### siRNA and transfections

The IPEC J2 cells were cultured to approximately 80% confluence in DMEM/F12 medium supplemented with 8% (v/v) FBS in 24-well plates. The cells were then transfected with 160 nM siRNA using Lipofectamine 2000 (Invitrogen) in Opti-MEM media (Gibco) according to the manufacturer’s instructions. After 6 h, the transfection medium was replaced with DMEM/F12 medium. Then the IPEC J2 cells were cultured with 8 mM PBA for 24 h. The cell lysates were harvested and analyzed by qRT-PCR. The small interfering RNA (siRNA) molecules targeting TLR2, TLR4, and a scrambled control were obtained from Shanghai Gene Pharma, and the sequences are presented in Table 2 (15).

**Table 2:** The sequences of siRNA

Names	Sequences(5'-3')	Reference
TLR2	CCA GAU CUU UGA GCU CCA UTT AUG GAG CUC AAA GAU CUG GTT	a
TLR4	GCA UGG AGC UGA AUU UCU ATT UAG AAA UUC AGC UCC AUG CTT	a
NC	UUC UCC GAA CGU GUC ACG UTT ACG UGA CAC GUU CGG AGA ATT	a

The sequences of siRNA. a (15).

### Plasmids transfections and luciferase reporter assays

The NF-κB p65 luciferase reporter plasmid (pNF-κB-Luc) and the internal-control plasmid-encoding Renilla luciferase (phRL-TK) were kindly provided by Prof. Guangxing Li (Northeast Agricultural University, Harbin, P. R. China). The IPEC J2 cells were co-transfected with 0.3 μg of pNF-κB-Luc and 0.1 μg of phRL-TK using the Lipofectamine 2,000 (Invitrogen) reagent in 24-well plates overnight at a density of 1×10^5^ cells/wells. After 6 h, the cells were treated with PBA at 8 mM and were cultured for 24 h continually. The cells stimulated by lipopolysaccharide (LPS) were used as a positive control, which usually activates the NF-κB pathway. The cell lysates were harvested and analyzed using the Dual-Luciferase^®^ Reporter Assay Kit (Promega, Madison, WI, USA). The luciferase activities were detected using a Promega GloMax 20/20 Luminous detector (Promega, China).

### Immunofluorescence assays

The IPEC J2 cells were seeded into 24-well plates and were treated with PBA (8 mM) and TSA (1 μM) for 24 h, washed with PBS, fixed with 200 μL 4% paraformaldehyde for 10 min, and quenched with 0.1 M glycine for 5 min at room temperature. Subsequently, the cells were permeabilized with 1% Triton X-100, and diluted into PBS for 10 min. After washing with PBS three times, the cells were then incubated at 37°C with a histone H3 mouse monoclonal antibody (1:200) for 45 min, washed three times, and were subsequently incubated with a TRITC-conjugated AffiniPure goat anti-mouse IgG(H+L) (1:200) for 30 min. Thereafter, the cells were washed with PBS and then stained with DAPI at 37°C for 10 min to detect the nuclei, and washed with PBS three times again. The fluorescence signals were visualized using a fluorescence microscope (Leica).

### Statistical analysis

All the results were expressed as the means ± SD. Differences between the groups were compared using an unpaired Student’s *t*-test or GLM (General Linear Model of Statistical Analysis System, SAS 9.4.2, 2000). Differences between the treatments were considered significant for *P* < 0.01.

## Results

### PBA facilitates endogenous HDP gene expression but does not enhance IL-6 production in IPEC J2 cells

**Fig. 1:**
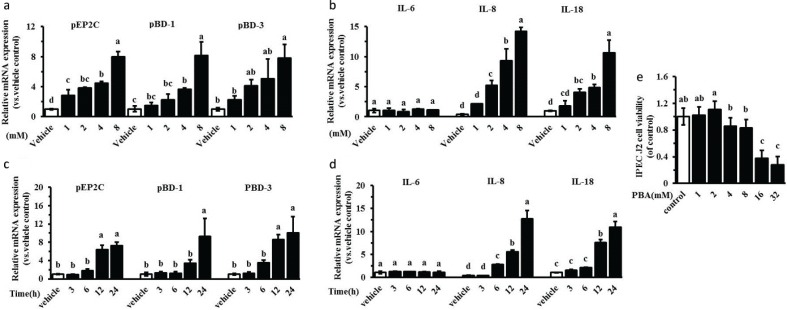
PBA upregulates endogenous HDPs gene expression. IPEC J2 cells were stimulated with 0 mM, 1 mM, 2 mM, 4 mM, and 8 mM PBA for 24 h (a, b) or 8 mM of PBA for 3 h, 6 h, 12 h, and 24 h (c, d). HDPs (pEP2C, pBD-1, pBD-3) and cytokines (IL-6, IL-8, IL-18) were analyzed by qRT-PCR. (e) IPEC J2 cells in a broad range of concentrations (0–32 mM) for 24 h, we used the MTT dye reduction assay to examine their toxicity. All data are expressed as the means ± SD. Letters with different superscripts are significantly different at *P* < 0.01, compared with vehicle.

Recent studies show that sodium 4-phenylbutyrate (PBA), an odorless derivative of butyrate sodium, is an even more potent inducer of cathelicidins in vitro than butyrate sodium (13). We investigated the expression of inducible genes encoding HDPs (pEP2C, pBD-1, pBD-3) and cytokines (IL-6, IL-8, IL-18) in the innate immune response by PBA. Our real-time PCR analyses indicated that HDP expression was markedly increased in a dose-dependent manner following a 24-h treatment with PBA in IPEC J2 cells (Fig. 1a). Similarly, the expression levels of IL-8 and IL-18 were dose-dependently induced by PBA (Fig. 1b). However, the mRNA level of the IL-6 gene was not affected. Furthermore, an obvious time-dependent induction of pEP2C, pBD-1, pBD-3, IL-8, and IL-18 was observed in the IPEC J2 cells, and the IL-6 expression was still not affected (Fig. 1c, 1d). Herein, the cytotoxicity was not significantly altered by PBA at concentrations ≤8 mM in the IPEC J2 cells, as assessed by the MTT assay (Fig. 1e). The concentration and time of PBA were selected at 8 mM and 24 hour respectively in the following trials.

### PBA-induced HDP gene expression via TLR2 in IPEC J2 cells

**Fig. 2:**
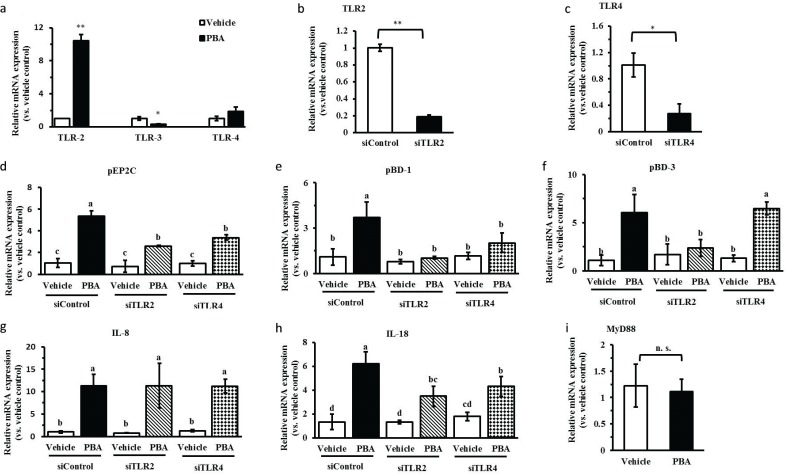
PBA-induced HDPs gene expression via TLR2. (a) Expressions of TLRs were determined by quantitative real-time PCR. (b, c) IPEC J2 cells were transfected with TLR2/4 siRNA to specifically silence TLR2/4 and then treated with PBA. TLR2/4(b, c), HDPs (pEP2C, pBD-1, pBD-3) (d, e, f), and cytokines (IL-8, IL-18) (g, h) gene expressions were analyzed by qRT-PCR. (i) IPEC J2 cells were treated with PBA for 24 h, by qRT-PCR to detect the expression of MyD88 mRNA. Letters with different superscripts are significantly different at *P* < 0.01, compared with vehicle.

TLRs mediate diverse signaling pathways, which recognize molecular-associated patterns of microorganisms. Intestinal epithelial cells express TLRs, and their activation leads to the production of anti- or pro-inflammatory cytokines contributing to inflammatory responses (17). Previous studies have shown that sodium butyrate activate TLR2 and then mediate HDP gene expression (16). In our studies, the expression of TLR2 was enhanced 10-fold by PBA, and the expression of TLR4 showed an increasing tendency but was not significant. However, the expression of TLR3 was significantly decreased by quantitative real-time PCR (Fig. 2a). We further evaluated the role of TLR2 or TLR4 in the gene regulation of encoding HDPs and cytokines by PBA. The IPEC J2 cells were transfected with a siRNA-targeting TLR2 or TLR4 to silence TLR2 or TLR4, respectively. Compared with the control siRNA, the results showed that TLR2 or TLR4 expression were reduced markedly following the transfection of TLR2/4 siRNA by qRT-PCR (Fig. 2b and 2c). Thereafter, we further analyzed the regulation changes of HDP expression by PBA after silencing TLR2 or TLR4. The results showed that even though the expression of pEP2C was still increased significantly by PBA, it was remarkably reduced in the cells treated with TLR2/4 siRNA, compared with the control siRNA by PBA (Fig. 2d). Most clearly, pBD-1, inducted by PBA, was dramatically and completely destroyed under the condition of silencing both TLR2 and TLR4 (Fig. 2d). Distinguishingly, TLR4 silencing did not effect pBD-3 mRNA expression, compared with the cells transfected with the negative control siRNA (Fig. 2f). Taken together, these data indicate that TLR2 silencing stopped or interfered with the upregulation of HDPs expression by PBA in IPEC J2 cells. Interestingly, TLR4 silencing had no effect on the pBD-3 induction by PBA, but the role of TLR4 signaling was similar to TLR2 with respect to the regulation of pEP2C and pBD-1 expression by PBA. Furthermore, the results further showed that the induction of IL-8 exhibited an obvious difference with the pBD-1, pBD-3, or pEP2C genes regulated by PBA, and the expression regulation of IL-8 by PBA was not altered after knocking down TLR2 or TLR4 completely (Fig. 2g). While the expression of IL-18 significantly declined in the IPEC J2 cells treated with TLR2 or 4 siRNA, the IL-18 expression was still elevated by PBA; this result was similar to when expression of the pEP2C induced by PBA was blocked by TLR2 or 4 silencing (Fig. 2h). The myeloid differentiation primary response gene adaptor molecule (MyD88) was involved in the TLR signaling pathways (18). Our results showed that PBA did not influence MyD88 mRNA levels (Fig. 2i), suggesting that PBA influenced signaling effectors during TLR activation but not MyD88.

### PBA activates the NF-k B signaling pathway in IPEC J2 cells and induces HDP gene expression

**Fig. 3:**
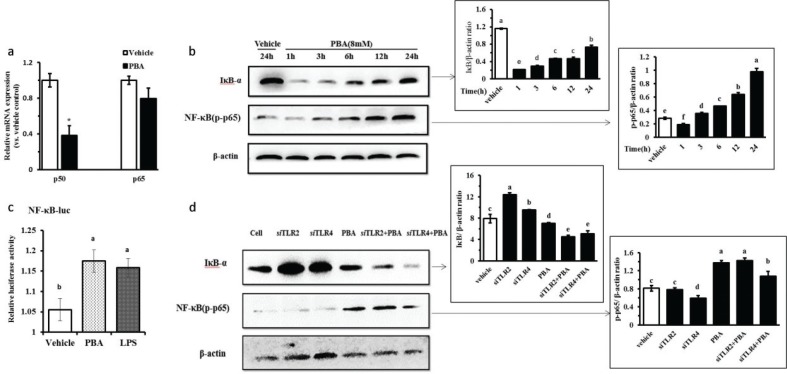
PBA-induced HDPs gene expression activates the NF-κB signaling pathway. (a) qRT-PCR analysis of p50 and p65 transcription in cells treated with 8 mM PBA for 24 h. (b) Immunoblot analysis of the phospho-NF-κB p65 protein and the IκB-α protein in cells pretreated or not for 0–24 h with 8 mM PBA. (c) IPEC J2 cells were co-transfected with the pNF-kB-Luc and the internal-control phRL-TK plasmids for 6 h, and were treated with PBA or LPS for 24 h. The cells were then harvested and analyzed by luciferase reporter assays. (d) The IPEC J2 cells were transfected with TLR2/4 siRNA to specifically silence TLR2/4 for 6 h and were then challenged with PBA at 8 mM for 24 h, and a Western blot was used to assess the protein levels of IκB-α and phospho-p65. A densitometric analysis of the IκB-α and phospho-p65 protein levels is represented as the mean ± SD from six independent experiments. Letters with different superscripts are significantly different at *P* < 0.01, compared with the vehicle.

Cytokine production mediated by TLR recognition and activation is usually dependent on the NF-κB pathway and MAPKs, and thus, we evaluated both signaling pathways after PBA stimulation in IPEC J2 cells. First, we took a luciferase reporter approach using a luciferase vector containing the NF-κB p65 initiation factor sequences, as previously reported by others. The IPEC J2 cells were co-transfected with the pNF-κB-Luc plasmid and the internal-control phRL-TK plasmid, and were then treated with PBA or LPS to address the effect on NF-κB translational activity. LPS is known as a positive stimulus of NF-κB. In agreement, we observed a strong expression of the NF-κB-regulated luciferase following PBA pretreatment compared to expression without treatment by the stimulant, and similar results were obtained in the IPEC J2 cells after stimulation with LPS (Fig. 3a). The classic NF-κB activation pathway is a multi-step process that involves several key proteins in inflammatory and immune responses and cellular proliferation. The most abundant form of NF-κB is a heterodimer of p50 and p65 (19). Our results showed a markedly reduced p50 expression but not p65 by qRT-PCR (Fig. 3b). Next, we observed a clear increase of NF-κB p65 phosphorylation in a time-dependent manner following PBA pretreatment (Fig. 3c). A crucial negative regulator that controls NF-κB activation is the inhibitor of κB (IκB); this inhibitor binds to p65 in the cytosol to block the nuclear translocation of the p65/p50 complex. Based on the IκB-α protein assays, we found that PBA eventually facilitated the proteasomal degradation of IκB-α in response to PBA treatment for 24 h, which freed p65/p50, allowing the entry of p65/p50 to the nucleus to activate gene expression. There was degradation at 24 h compared with the vehicle without pretreatment; however, it is interesting that an increased expression of IκB-α protein from 6 h to 24 h under the internal reference calibration were observed (Fig. 3c). Collectively, our results establish that PBA upregulates HDP expression and activates the NF-κB pathway.

To identify whether TLR2/4 mediated the activation of the NF-κB signaling pathway by PBA, the IPEC J2 cells were transfected with a siRNA-targeted TLR2/4 to specifically silence TLR2/4 and were then challenged with PBA, and the protein levels of IκB-α and phospho-p65 were assessed. The data showed that both TLR2 and TLR4 silencing still markedly facilitated the degradation of IκB-α protein compared with the non-silencing control after PBA treatment in the IPEC J2 cells. However, interestingly, IκB-α protein synthesis increased significantly when both TLR2 and TLR4 were silenced alone without treatment by PBA. It was observed that TLR4 silencing slightly influenced p65 phosphorylation induced by PBA, but no effect by TLR2 (Fig. 3d). The above results indicate that TLR2 and TLR4 silencing did not inhibit the IκB-α degradation induced by PBA indirectly. However, TLR4 silencing decreased the phosphorylation of p65 but not completely.

### Histone modification occurs while PBA induces the HDP gene expression increase in IPEC J2 cells

**Fig. 4:**
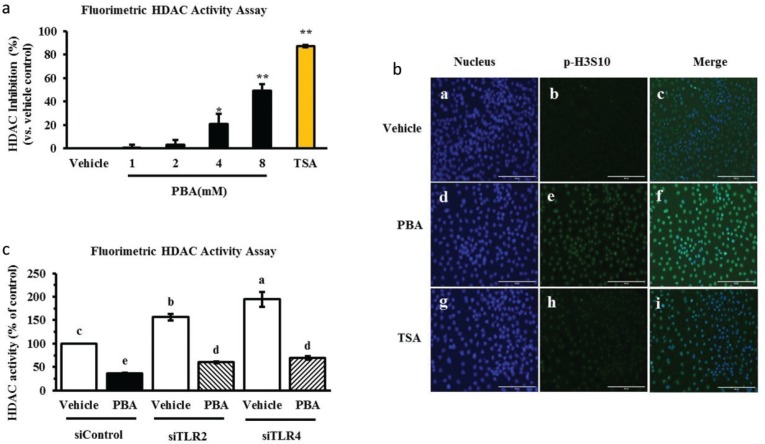
PBA induces HDP gene expression upon histone modification. (a) HDAC activity was determined. The cells were incubated with increasing concentrations of PBA, and TSA was used as a reference. (b) An immunofluorescence analysis of phospho-histone H3 in the IPEC J2 cells, which were treated with PBA and TSA for 24 h. (c) The IPEC J2 cells were transfected with TLR2/4 siRNA before a treatment with PBA, and then HDAC activity was detected. Letters with different superscripts are significantly different at *P* < 0.01 compared with the vehicle.

Phenylbutyrate is known as a reversible inhibitor of class I and II HDACs. It is considered a first generation HDAC inhibitor due to its non-specific inhibitory effect. Moreover, PBA exerts its effects in relatively high, millimolar working concentrations, and the effects are pleiotropic. Several studies suggest that the HDAC inhibitor TSA or butyrate significantly impacts the induction of antimicrobial peptide gene expression and requires the acetylation of histones H3 at several lysine residues (8, 9). We therefore investigated whether PBA behaves as an HDAC inhibitor in IPEC J2 cells. By HDAC activity detection, we identified a significant dose-dependent manner of HDAC activity inhibition efficiency with PBA in the IPEC J2 cells, and TSA (1μM) was a positive control of the HDAC inhibition (Fig. 4a). In addition, the histone H3 phosphorylation levels were observed by immunofluorescence. There was a strong increase in the fluorescence intensity of the phosphorylation marker following PBA and TSA pretreatment for 24 h compared with the control, which was without treatment in the IPEC J2 cells. Together, these results indicate that PBA regulated histone modification including deacetylation and phosphorylation in IPEC J2 cells, as well as upregulated HDP expression and has no effect on IL-6 expression (Fig. 4b); these results are a reminder that epigenetic pharmacology should be achieved to induce epithelial host defense.

In addition, as the above results show, TLR2 and TLR4 silencing affected IκB-α protein and p65 phosphorylation levels and regulated HDP gene expression induced by PBA. It is interesting whether TLR2 or TLR4 influences HDAC activity with or without PBA treatment. First, IPEC J2 cells were transfected with an siRNA-targeted TLR2 or TLR4 for 6 h to specifically silence TLR2 or TLR4, and then, the cells were treated with PBA for 24 h. As revealed by HDAC activity detection assay, our results indicated that HDAC activity inhibition by PBA was not slow down after TLR2 or TLR4 silencing, interestingly, TLR2 and TLR4 silencing alone significantly enhanced HDAC activation which had the opposite effect on HDAC activation with PBA (Fig. 4c). Taken together, these results suggest that PBA improved HDP gene expression upon histone modification.

### Activation of the MAPK pathway is necessary for PBA-mediated HDP upregulation

**Fig. 5:**
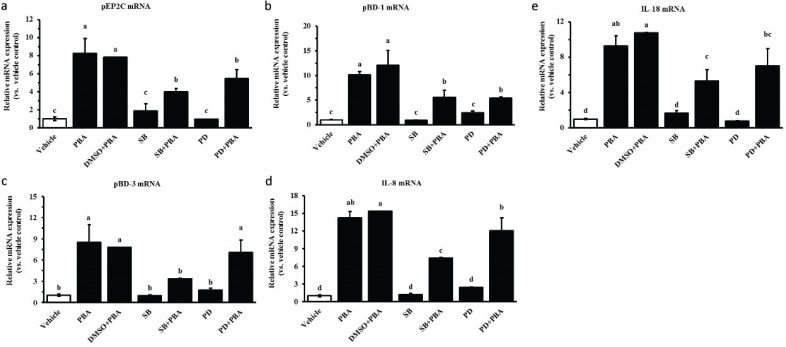
The effect of MAPK inhibitors on the upregulation of PBA-mediated HDP expression. IPEC J2 cells were pretreated with the p38 MAPK inhibitor SB203580 and the ERK1/2 inhibitor PD98059 for 6 h before incubation with PBA at 8 mM for 24 h. qRT-PCR was used to detect the gene expression of (a) pEP2C, (b) pBD-1, (c) pBD-3, (d) IL-8, and (e) IL-18. Letters with different superscripts are significantly different at *P* < 0.01 compared with the vehicle.

Previous studies indicate that PBA-induced CAMP gene expression is attenuated by MAPK inhibitors (13). We therefore analyzed HDP expression in IPEC J2 cells treated with the specific inhibitors of the MAPK pathways by qRT-PCR. The IPEC J2 cells were pretreated with the p38 MAPK inhibitor SB203580 and the ERK1/2 inhibitor PD98059 for 6 h before incubation with PBA at 8 mM for 24 h. DMSO was the solvent of the reagent. As shown in Fig. 5a, Fig. 5b, and Fig. 5c, the inhibitors SB203580 and PD98059 significantly reduced PBA-induced pEP2C and pBD1 gene expressions but they were not inhibited completely. However, PD98059 failed to inhibit the pBD-3 induction by PBA determined at the mRNA level. The MAPK pathway also plays a critical role in intracellular cytokine production. In addition, PBA influences the activation of the cytokines IL-8 and IL-18 in IPEC J2 cells. To test whether the MAPK pathway is involved in the induction of cytokines by PBA in the IPEC J2 cells, further studies were performed to detect the effects of the inhibitors SB203580 and PD98059 on IL-8 and IL-18 mRNA expression before PBA treatment. Interestingly, SB203580 markedly suppressed the production of IL-8 and IL-18 induced by PBA. In contrast, there was no significant change in the IL-8 and IL-18 mRNA levels after a pretreatment with PD98059 compared with a treatment with PBA alone (Fig. 5d, 5e). Overall, the data from the above experiment demonstrate the relevance of host defense cytokines and the MAPK pathway in innate host defense. The p38 MAPK and ERK1/2 pathway may have partially contributed to the upregulation of the HDPs pEP2C, pBD-1, and pBD-3 mediated by PBA, but the ERK1/2 pathway did not influence pBD-3 upregulation inducted by PBA in the IPEC J2 cells.

### EGFR is a critical factor for PBA-mediated HDP upregulation

**Fig. 6:**
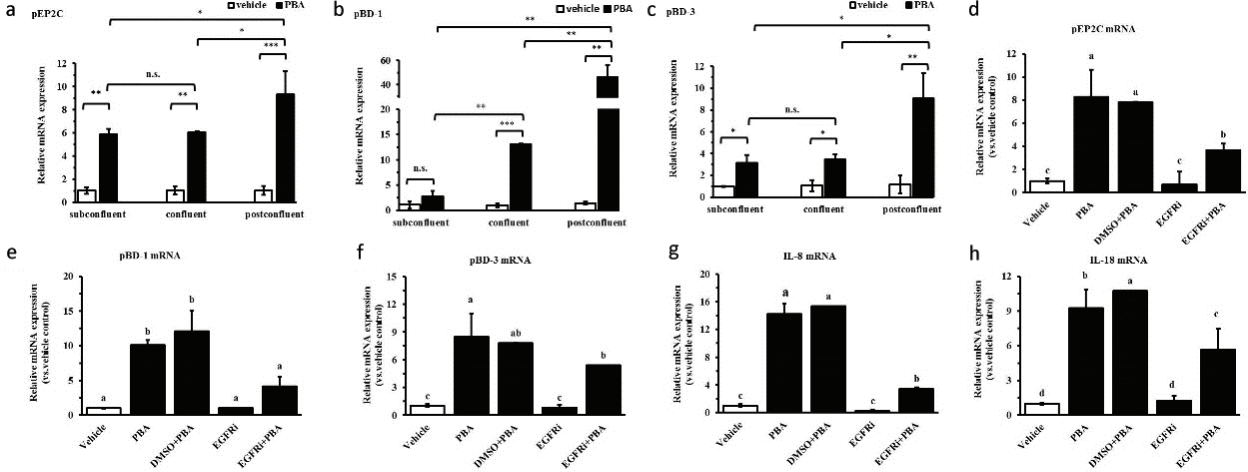
The effect of cell density or EGFR on the upregulation of PBA-mediated HDP expression. (a–c) qRT-PCR analysis of pEP2C, pBD-1, and pBD-3 transcription at different cell densities treated with 8 mM PBA. (d–h) The IPEC J2 cells were pretreated with the EGFR inhibitor Gefitinib and were then treated with PBA. Letters with different superscripts are significantly different at *P* < 0.01, compared with the vehicle.

IPEC J2 cells possess the typical feature of growth polarity and form tight junctions during cell growth, differentiation, survival, and movement in response to different degrees of confluence. Previous studies report that differences in the EGFR transcript expression levels at different degrees of confluence affect the production of antimicrobial peptides (20). Therefore, different degrees of confluence were mimicked by culturing the cells at different cell densities, including subconfluent, confluent, and post-confluent; at a subconfluent cell density and at a confluent cell density, there was relatively little inducible pEP2C, pBD-1, and pBD-3 mRNA expression compared with the post-confluent cell density, although the HDPs were markedly increased at any confluences; pEP2C, pBD-1, and pBD-3 mRNA increased 10-, 50-, and 10-fold, respectively, for cells stimulated with PBA at a post-confluent density (Fig. 6a, 6b, 6c). It is reported that EGFR is expressed at dramatically higher levels in post-confluent density than in subconfluent and confluent conditions, and the signaling pathways showed a switch in the post-confluent cells (20). To understand the correlation of EGFR in the intestinal epithelial cells, IPEC J2 cells were pretreated with the gefitinib, an EGFR inhibitor, for 6 h and were treated with 8 mM PBA. DMSO was in control of the solvent of the gefitinib. The results showed that the increased mRNA expression of pEP2C, pBD-1, and pBD-3 by PBA were downregulated in the presence of gefitinib compared with the PBA treatment alone by qRT-PCR assay (Fig. 6d 6e, 6f). In addition, gefitinib caused a significant reduction in IL-8 or IL-18 expression compared to the PBA treatment alone (Fig. 6g, 6h). The above results show that EGFR is a critical factor governing the regulation of HDP expression by PBA; this regulation is consistent with the trends of HDP expression regulation by the PBA between the different degrees of confluence, as expected.

## Discussion

The establishment and maintenance of epithelial homeostasis were contributed to various actors in the intestinal tract. HDPs, as an essential component of innate immunity, have the potential to regulate and improve intestinal barrier function in animal health and productivity (21, 22). Oral supplementation of HDPs-induced compounds show promise in preventing and controlling infections in humans and several animal species (7). HDPs and cytokine genes are generally considered to be synchronously expressed in innate immune response to diverse pathogenic microorganism stimuli. *Staphylococcus aureus* or lipopolysaccharide (LPS) induces the expression of several HDPs including bovine β-defensin 1 and bovine neutrophil β-defensin 4, after infection (23). In our studies, PBA, which is an odorless derivative of butyric acid naturally produced by colonic bacteria fermentation, increased the endogenous HDP gene expression of pEP2C, pBD-1, and pBD-3 and the cytokine IL-8 and IL-18 production in IPEC J2 cells. However, PBA had no effect on the expression level of pro-inflammatory IL-6. This result is exactly what the difference between nutrients and pathogenic microorganisms exposed to the surface of host cells is shown to be on innate immunity. This work suggests that PBA may be a potential functional feed additive to achieve the induction of epithelial antimicrobial defenses while limiting the deleterious risk of an inflammatory response.

As we know, this mechanism occurring in jejunum epithelial cells is orchestrated between HDP gene expression regulation and exogenous stimulus mainly through signaling pathways, which result in the recognition of a receptor, chromatin histone modification, the activation of key signaling factors, and so on. Therefore, it is indispensable to further investigate the mechanism between HDP gene expression and PBA in jejunum epithelial cells. TLRs are generally activated in response to a diverse array of microbial products. Human corneal epithelial cells (HCECs) express TLR2, which responds to *Staphylococcus aureus* infection through the expression and secretion of pro-inflammatory cytokines and β-defensin-2 (hBD2) (24). In addition, human tracheobronchial epithelial cells respond to bacterial lipopeptide in a TLR2-dependent manner with the induction of mRNA and protein of the antimicrobial peptide human defensin-2 (25). In our studies, PBA also activated the TLR2 and inhibited TLR3 expression in IPEC J2 cells. Moreover, TLR2 silencing weakened the ability of HDPs expression induction by PBA; this outcome was similar to the result of sodium butyrate in porcine kidney cells, but the activation ability of PBA in IPEC J2 cells was less than sodium butyrate in porcine kidney cells (16). The production of pEP2C, pBD-1, and pBD-3 in IPEC J2 cells stimulated with PBA also occurred in a TLR4-dependent pathway although TLR4 was not markedly activated. PBA possesses the ability to regulate HDP expression and PEP2C, pBD-1, and pBD-3 are well-known as HDPs for their antimicrobial activity against a broad range of bacterial, fungal, and viral pathogens; TLRs are the viral recognition receptors of pathogenic microorganisms (4), however, in our results, which showed a regulatory role in the HDPs expression regulation by PBA.

Intestinal epithelial cells have long been known to provide a source of inflammatory cytokines and chemokines (26), and they also gather different kinds of HDPs. Recent studies demonstrate that HDPs function as immunomodulatory mediators and antimicrobial agents through either direct chemotactic activity or the upregulation of several cytokines and chemokines in various cell types. Cathelicidin LL-37 not only favorably induces IL-8 expression and secretion in human gingival epithelial cells (27) but also increases IL-18 mRNA expression in keratinocytes (28). Likewise, we found that PBA upregulated endogenous HDPs and cytokine production in IPEC J2 cells. We hypothesized that HDPs and cytokines were not synchronously expressed with different regulatory rules; however, a TLR2/4-dependent activation of epithelial cells induced cytokine IL-18 gene expression, and HDPs. In addition, IL-8 gene expression was not affected by TLR2/4. It remains to be determined which signaling pathways are responsible for TLR2/4-dependent HDP and cytokine production and which other signaling pathways are activated.

The NF-κB pathway, as a hub of regulation in the host immune defense between many exogenous stimuli, activates host immunity, particularly the expression of regulatory cytokines. In our studies, PBA modulates NF-κB signaling in IPEC J2 cells; this modulation is dependent on the enhancement of NF-κB p65 phosphorylation and IκB α degradation. Furthermore, we found that TLR2 or TLR4 silencing did not affect PBA-induced NF-κB p65 phosphorylation and IκB degradation. MyD88, a primary adaptor molecule of TLRs, was not affected by PBA, indicating that the PBA effect on HDP production was different than the pathogen-associated molecular pattern (PAMPs). In this case, the results indicated that PBA-induced HDP increase was related to the TLR2/4 and NF-κB signaling pathway; p65 (NF-κB3) and p50 (NF-κB1) are two key subunits of the NF-κB pathway, and p50 lacks a transcriptional activation domain, which induces its downstream target gene expression by interacting with other transcription factors or transcription co-activators (19, 29). In our studies, a dramatic decrease in p50 expression was present after PBA treatment, which suggests the NF-κB pathway activation in the IPEC J2 cells. In agreement, lower p50 levels were beneficial for HDP expression. The increase in both p65 phosphorylation in a time-dependent manner and the NF-κB luciferase activity following PBA pretreatment together suggested that NF-κB was activated by PBA in the IPEC J2 cells. Interestingly, an apparent increase in IκB α protein levels was observed in a time-dependent manner after PBA treatment, but there was significant degradation compared with the untreated control cells. Previous studies show that trichostatin A potentiates tumor necrosis factor (TNF) α-elicited NF-κB activation by histone deacetylase inhibitor (HDACi) and delays IκB α cytoplasmic reappearance (19, 30). PBA is also a HDAC inhibitor, which suggested to us that PBA induced HDP gene expression via delaying IκB α synthesis and then activating the NF-κB pathway.

Acetylation is a pivotal post-translational modification of numerous proteins, such as histones and transcription factors, including NF-κB. Histone acetylation and deacetylation modifications play a crucial role in the chromatin structure, cellular function, and transcriptional regulation of gene expression (30, 31). Two enzyme families with opposite activities are crucial regulators of gene expression. Histone acetyltransferase (HAT) acts in a positive manner and HDAC acts in a negative manner. NF-κB functions are regulated by post-translational modifications, including phosphorylation and acetylation (32). Several studies show that HDAC 3 induces the NF-κB p65 subunit deacetylation, leading to the repression of its transcriptional activity (33). In addition, we addressed HDAC inhibitors as an approach to attenuate inflammatory responses and their potential as novel therapeutics (33). In our study, a significant dose-dependent inhibition of HDAC activity was detected after PBA incubation. Furthermore, PBA-mediated HDAC inhibition activated an alternative pathway, inducing H3S10 phosphorylation. The phosphorylation of H3S10, as well as the acetylation of histone H3 lysines, is highlighted in the current model as discrete modifications promoting chromatin remodeling at the promoter of specific innate immune genes, allowing the precise recruitment of NF-κB (9, 34). Herein, the delay of IκB α protein synthesis seems to be due to impairing of the recruitments of p65 phosphorylation, but not histone H3 on Ser10 phosphorylation (30). PBA could also be an HDACi, as it enhances HDPs and then attenuates inflammatory responses in IPEC J2 cells. Interestingly, TLR2 and TLR4 silencing did not reverse adjust the inhibition of HDAC activity by PBA. Moreover, both TLR2 and TLR4 silencing alone increased the HDAC ability in IPEC J2 cells; this result was consistent with the control of PBA increasing TLR2 and TLR4 expression and then the inhibition of the HDAC activity, as in the results above from our studies.

In a previous study, the canonical phosphorylation of histone H3 occurred through the activation of the MAPK signaling pathway, and both ERK and p38 kinases induced the phosphorylation of H3S10 at the promoter of the activated genes (35). In addition, ERK and MAPK signaling pathways are involved in cathelicidin gene expression induced by PBA (13). In this study, we observed that both the p38 MAPK inhibitor SB203580 and the ERK1/2 inhibitor PD98059 weakened the pEP2C and pBD-1 expression induced by PBA; this result had a distinct effect on blocking the induction of pBD-3. In addition, ERK1/2 signal blockade had no effect on the induction of pBD-3, IL-8, and IL-18 by PBA; this outcome indicated to us that the signaling pathway of both the p38 and ERK1/2 signaling pathways participated in the regulatory mechanism of pEP2C, pBD-1, and pBD-3, but were not identical. In addition, unexpectedly, the degree of confluence significantly improved the regulatory ability of PBA on HDP expression in the IPEC J2 cells. In a previous report, the degree of confluence also changed the regulatory ability of sodium butyrate on HDP expression in sodium butyrate in porcine kidney cells, but the trend was just in contrast with this study. As reported, epidermal growth factor receptor (EGFR) expression levels increased as the degree of confluence increased (20). EGFR was also critical in the regulation of cathelicidin expression (12). Presumably, EGFR may play a role in the process of PBA-regulated HDP expression in IPEC J2 cells. As expected, inhibition of EGFR with a specific inhibitor significantly reduced PBA-increased HDPs gene expression. Moreover, in porcine Intestinal epithelial cells, host defense peptides regulation maybe utilized different signal transduction pathways or a switch in signaling pathways with the altered degrees of confluence, including sub confluent, confluent, and post-confluent (20).

**Fig. 7:**
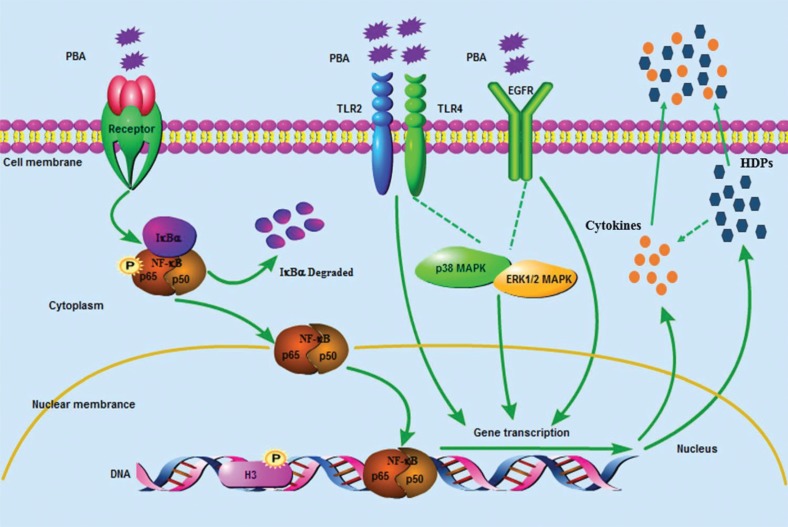
The signaling pathway of defensins and interleukins gene expression is regulated by PBA in porcine intestinal epithelial cells. Sodium phenyl butyrate (PBA) activated the NF-κB pathway via the phosphorylation of p65 and IκB α synthesis delayed and degraded. TLR2, TLR4, and EGFR were required for the PBA-mediated up-regulation of the HDPs. Meanwhile, histone deacetylase (HDAC) inhibition and an increased phosphorylation of histone H3 on serine S10 also occurred in PBA-induced HDP expression independently on TLR2 and TLR4. Furthermore, p38-MAPK suppressed PBA-induced pEP2C, pBD-1, pBD-3, IL-8, and IL-18 expression, and ERK1/2 abolished the regulation of pEP2C and pBD-1. In conclusion, HDPs and interleukins expression were regulated by PBA via a complex route system.

To conclude, as shown in Figure 7, PBA regulated HDPs and interleukins expression closely via a complex route system.

## Funding

This work was financially supported by the National Natural Science Foundation of China (31472104, 31672434), Natural Science Foundation of Heilongjiang Province (C2018028), the China Postdoctoral Science Foundation (2017M621237), Postdoctoral Foundation in Heilongjiang Province (LBH-Z17013), and the China Agriculture Research System (CARS-35).

## References

[1] O’Neill J The review on antimicrobial resistance: tracking drug resistant infections globally. Wellcome Trust and the Department of Health of UK Government 2016.

[2] Hancock REW, Haney EF, Gill EE The immunology of host defence peptides: beyond antimicrobial activity. Nat Rev Immunol 2016; 16: 321. 27087664 10.1038/nri.2016.29

[3] Zhang LJ, Gallo RL Antimicrobial peptides. Curr Biol Cb 2016; 26: R14. 26766224 10.1016/j.cub.2015.11.017

[4] Sang Y, Blecha F Porcine host defense peptides: expanding repertoire and functions. Dev Comp Immunol 2009; 33: 334–43. 18579204 10.1016/j.dci.2008.05.006

[5] Choi MK, Le MT, Nguyen DT, Choi H, Kim W, Kim JH, et al. Genome-level identification, gene expression, and comparative analysis of porcine ß-defensin genes. Bmc Genet 2012; 13: 98. 23150902 10.1186/1471-2156-13-98 PMC3499285

[6] Zeng X, Sunkara LT, Jiang W, Bible M, Carter S, Ma X, et al. Induction of porcine host defense peptide gene expression by short-chain fatty acids and their analogs. Plos One 2013; 8: e72922. 24023657 10.1371/journal.pone.0072922 PMC3758276

[7] Lyu W, Curtis AR, Sunkara LT, Zhang G Transcriptional regulation of antimicrobial host defense peptides. Curr Protein Pept Sci 2015; 16: 672–9. 26122785 10.2174/1389203716666150630133432

[8] Xiong H, Guo B, Gan Z, Song D, Lu Z, Yi H, et al. Butyrate upregulates endogenous host defense peptides to enhance disease resistance in piglets via histone deacetylase inhibition. Sci Rep 2016; 6: 27070. 27230284 10.1038/srep27070 PMC4882515

[9] Fischer N, Sechet E, Friedman R, Aurélien Amiot, Sobhani I, Nigro G, et al. Histone deacetylase inhibition enhances antimicrobial peptide but not inflammatory cytokine expression upon bacterial challenge. P Natl Acad Sci USA 2016; 113: 201605997. 10.1073/pnas.1605997113 PMC4889416 27162363

[10] Coussens AK, Wilkinson RJ, Martineau AR Phenylbutyrate is bacteriostatic against mycobacterium tuberculosis and regulates the macrophage response to infection, synergistically with 25-Hydroxy-Vitamin D_3_. Plos Pathogens 2015; 11: e1005007. 26133770 10.1371/journal.ppat.1005007 PMC4489717

[11] Merzvinskyte R, Treigyte G, Savickiene J, Magnusson KE, Navakauskiene R Effects of histone deacetylase inhibitors, sodium phenyl butyrate and vitamin B3, in combination with retinoic acid on granulocytic differntiation of human promyelocytic leukemia HL-60 cells. Ann Ny Acad Sci 2006; 1091: 356–67. 17341628 10.1196/annals.1378.080

[12] Kulkarni NN, Yi Z, Huehnken C, Agerberth B, Gudmundsson GH Phenylbutyrate induces cathelicidin expression via the vitamin D receptor: Linkage to inflammatory and growth factor cytokines pathways. Mol Immunol 2014; 63: 530–39. 10.1016/j.molimm.2014.10.007 25458314

[13] Kulkarni NN, Yi Z, Huehnken C, Agerberth B, Gudmundsson GH Phenylbutyrate induces antimicrobial peptide expression. Antimicrob Agents Chemother 2009; 53: 5127. 19770273 10.1128/AAC.00818-09 PMC2786349

[14] Mariani V, Palermo S, Fiorentini S, Lanubile A, Giuffra E Gene expression study of two widely used pig intestinal epithelial cell lines: IPEC-J2 and IPI-2I. Vet Immunol Immunopathol 2009; 131: 278–84. 19446887 10.1016/j.vetimm.2009.04.006

[15] Cao L, Ge X, Gao Y, Ren Y, Ren X, Li G Porcine epidemic diarrhea virus infection induces NF-κB activation through the TLR2, TLR3 and TLR9 pathways in porcine intestinal epithelial cells. J Gen Virol 2015; 96: 1757. 25814121 10.1099/vir.0.000133

[16] Dou X, Han J, Song W, Dong N, Xu X, Zhang W, et al. Sodium butyrate improves porcine host defense peptide expression and relieves the inflammatory response upon toll-like receptor 2 activation and histone deacetylase inhibition in porcine kidney cells. Oncotarget 2017; 8: 26532. 28460447 10.18632/oncotarget.15714 PMC5432277

[17] Fukata M, Vamadevan AS, Abreu MT Toll-like receptors (TLRs) and Nod-like receptors (NLRs) in inflammatory disorders. Semin Immunol 2009; 21: 242–53. 19748439 10.1016/j.smim.2009.06.005

[18] Kamdar K, Nguyen V, Depaolo RW Toll-like receptor signaling and regulation of intestinal immunity. Virulence 2013; 4: 207–12. 23334153 10.4161/viru.23354 PMC3711978

[19] Quivy V, Van LC Regulation at multiple levels of NF-kappaB-mediated transactivation by protein acetylation. Biochem Pharmacol 2004; 68: 1221. 15313420 10.1016/j.bcp.2004.05.039

[20] Johnston A, Gudjonsson JE, Aphale A, Guzman AM, Stoll SW, Elder JT EGFR and IL-1 signaling synergistically promote keratinocyte antimicrobial defenses in a differentiation-dependent manner. J Invest Dermatol 2011; 131: 329–37 20962853 10.1038/jid.2010.313 PMC3094455

[21] Robinson K, Deng Z, Hou Y, Zhang G Regulation of the intestinal barrier function by host defense peptides. Front Vet Sci 2015; 2: 57. 26664984 10.3389/fvets.2015.00057 PMC4672242

[22] Peterson LW, Artis D Intestinal epithelial cells: regulators of barrier function and immune homeostasis. Nat Rev Immunol 2014; 14: 141. 24566914 10.1038/nri3608

[23] Alva-Murillo N, Téllez-Pérez AD, Sagrero-Cisneros E, López-Meza JE, Ochoa-Zarzosa A Expression of antimicrobial peptides by bovine endothelial cells. Cell Immunol 2012; 280: 108–12. 23298865 10.1016/j.cellimm.2012.11.016

[24] Kumar A, Zhang J, Yu FX Toll-like receptor 2-mediated expression of β-defensin-2 in human corneal epithelial cells. Microb Infect 2006; 8: 380–9. 10.1016/j.micinf.2005.07.006 PMC2666383 16242370

[25] Hertz CJ, Wu Q, Porter EM, Zhang YJ, Weismüller KH, Godowski PJ, et al. Activation of Toll-like receptor 2 on human tracheobronchial epithelial cells induces the antimicrobial peptide human beta defensin-2. J Immunol 2003; 171: 6820–6. 14662888 10.4049/jimmunol.171.12.6820

[26] Stadnyk AW Intestinal epithelial cells as a source of inflammatory cytokines and chemokines. J Canadien de Gastroenterologie 2002; 16: 241–6. 10.1155/2002/941087 11981577

[27] Montreekachon P, Nongparn S, Sastraruji T, Khongkhunthian S, Chruewkamlow N, Kasinrerk W, et al. Favorable interleukin-8 induction in human gingival epithelial cells by the antimicrobial peptide LL-37. Asian Pacific Journal of Allergy & Immunology 2014; 32: 251–60. 25268344 10.12932/AP0404.32.3.2014

[28] François Niyonsaba, Ushio H, Nagaoka I, Okumura K, Ogawa H The human beta-defensins (-1, -2, -3, -4) and cathelicidin LL-37 induce IL-18 secretion through p38 and ERK MAPK activation in primary human keratinocytes. J Immunol 2005; 175: 1776. 16034119 10.4049/jimmunol.175.3.1776

[29] Luo MC, Zhou SY, Feng DY, Xiao J, Li WY, Xu CD, et al. Runt-related transcription factor 1 (RUNX1) binds to p50 in macrophages and enhances TLR4-triggered inflammation and septic shock. J Biol Chem 2016; 291: 22011. 27573239 10.1074/jbc.M116.715953 PMC5063984

[30] Horion J, Gloire G, El Mjiyad N, Quivy V, Vermeulen L, Vanden Berghe W, et al. Histone deacetylase inhibitor trichostatin A sustains sodium pervanadate-induced NF-kappaB activation by delaying ikappaBalpha mRNA resynthesis: comparison with tumor necrosis factor alpha. J Biol Chem 2007; 282: 15383–93. 17409387 10.1074/jbc.M609166200

[31] Furumai R, Ito A, Ogawa K, Maeda S, Saito A, Nishino N, et al. Histone deacetylase inhibitors block nuclear factor-κB-dependent transcription by interfering with RNA polymerase II recruitment. Cancer Science 2011; 102: 1081. 21299717 10.1111/j.1349-7006.2011.01904.x

[32] Wollebo HS, Bellizzi A, Cossari DH, Safak M, Khalili K, White MK Epigenetic regulation of polyomavirus JC involves acetylation of specific lysine residues in NF-κB p65. J Neurovirol 2015; 21: 679–87. 25791343 10.1007/s13365-015-0326-2 PMC4575817

[33] Leus NGJ, Zwinderman MRH, Dekker FJ Histone deacetylase 3 (HDAC 3) as emerging drug target in NF-κB-mediated inflammation. Curr Opin Chem Biol 2016; 33: 160–8. 27371876 10.1016/j.cbpa.2016.06.019 PMC5019345

[34] Saccani S, Pantano S, Natoli G p38-dependent marking of inflammatory genes for increased NF-|[kappa]|B recruitment. Nat Immunol 2002; 3: 69. 11743587 10.1038/ni748

[35] Clayton AL, Mahadevan LC MAP kinase-mediated phosphoacetylation of histone H3 and inducible gene regulation. Febs Lett 2003; 546: 51–8. 12829236 10.1016/s0014-5793(03)00451-4

